# Prevalence and risk factors of subclinical hypothyroidism in postmenopausal women: A cross-sectional study

**DOI:** 10.6026/973206300212241

**Published:** 2025-07-31

**Authors:** Chakresh Jain, Nishi Mishra, Sarita Singh, Arunendra Nirat

**Affiliations:** 1 Department of Community Medicine, Shyam Shah Medical College, Rewa, Madhya Pradesh, India; 2 Department of Obstetrics & Gynecology, Government Medical College, Santa, Madhya Pradesh, India; 3 Department of Obstetrics & Gynecology, Shyam Shah Medical College, Rewa, Madhya Pradesh, India; 4 Department of Community Medicine, Nandkumar Singh Chouhan Government Medical College, Khandwa, Madhya Pradesh, India

**Keywords:** Subclinical hypothyroidism, postmenopausal women, thyroid-stimulating hormone (TSH), risk factors, prevalence

## Abstract

The prevalence and associated risk factors of subclinical hypothyroidism among 142 postmenopausal women aged 45-70 years. Thyroid profiles were evaluated using serum TSH and free T4 levels, identifying subclinical hypothyroidism in 22.5% of participants. Higher BMI, dyslipidemia, and hypertension showed significant associations with thyroid dysfunction. Subclinical hypothyroidism was more common among women within 5-10 years of menopause. These findings underscore the need for routine thyroid screening in postmenopausal women.

## Background:

Subclinical hypothyroidism (SCH) is characterized by elevated serum thyroid-stimulating hormone (TSH) levels with normal free thyroxine (free T4) and often remains asymptomatic [1]. It is more prevalent in women and tends to increase with age, particularly after menopause [2]. Postmenopausal women are at heightened risk due to hormonal changes that may affect thyroid function and metabolism [3]. SCH is clinically important as it can progress to overt hypothyroidism and is associated with cardiovascular risks, metabolic disturbances and reduced quality of life [4]. Despite its significance, data on the prevalence and risk factors for SCH in postmenopausal women remain limited, especially in diverse populations [5]. Identifying these factors can guide early detection and management to prevent complications [6]. Therefore, it is of interest to determine the prevalence of SCH and evaluate its association with demographic, clinical, and metabolic parameters in postmenopausal women.

## Materials and Methods:

This cross-sectional study was conducted over 10 months at a tertiary care center and included 142 postmenopausal women aged 45 to 70 years. Menopause was defined as the absence of menstruation for at least 12 months. Participants with known thyroid disease, on thyroid medication, or with acute illness were excluded. After obtaining informed consent, detailed demographic and clinical data were collected, including age, duration since menopause, BMI, blood pressure, and medical history. Fasting blood samples were collected to measure serum TSH, free T4, fasting glucose, and lipid profile using standardized laboratory methods. Subclinical hypothyroidism was defined as TSH levels between 4.5 and 10 mIU/L with normal free T4. Statistical analysis was performed using SPSS version 26. Continuous variables were expressed as mean ± SD and compared using t-tests or Mann-Whitney U tests. Categorical variables were analyzed using chi-square tests. Logistic regression analysis was conducted to identify independent risk factors for SCH, with p < 0.05 considered statistically significant.

## Results:

In this study of 142 postmenopausal women, the prevalence of subclinical hypothyroidism (SCH) was 22.5%. Women with SCH had significantly higher BMI, blood pressure, and altered lipid profiles compared to euthyroid women. Several clinical and biochemical parameters were associated with SCH, highlighting important risk factors in this population. Table 1 (see PDF) shows Women with SCH had a significantly higher mean age and longer duration since menopause compared to euthyroid women, indicating age and menopausal duration as important factors. Table 2 (see PDF) shows Systolic and diastolic blood pressures were significantly elevated in women with SCH, highlighting increased cardiovascular risk. Table 3 (see PDF) shows the prevalence of hypertension and dyslipidemia was significantly higher in the SCH group, indicating metabolic comorbidities associated with thyroid dysfunction. Table 4 (see PDF) shows Serum TSH levels were significantly elevated in the SCH group, while free T4 remained within normal limits, confirming the diagnosis. Table 5 (see PDF) shows Women with SCH showed significantly higher total cholesterol and LDL cholesterol levels compared to euthyroid women, underscoring a pro-atherogenic lipid profile. Table 6 (see PDF) shows Fasting blood glucose levels were higher in SCH women but did not reach statistical significance. Table 7 (see PDF) shows Duration since menopause longer than 5 years was associated with a higher risk of SCH, indicating menopausal hormonal changes may influence thyroid function. Table 8 (see PDF) shows Body mass index (BMI) above 27 kg/m^2^ was significantly associated with SCH prevalence. Table 9 (see PDF) shows Multivariate logistic regression identified BMI > 27 kg/m^2^, hypertension, and duration since menopause > 5 years as independent predictors of SCH. Table 10 (see PDF) shows the prevalence of dyslipidemia increased significantly with rising TSH levels, suggesting a dose-response relationship.

## Discussion:

This cross-sectional study found a 22.5% prevalence of subclinical hypothyroidism (SCH) among postmenopausal women, consistent with prior reports indicating increased thyroid dysfunction risk in this group. The study identified key risk factors including higher BMI, hypertension, and longer duration since menopause, highlighting the interplay between metabolic and hormonal changes after menopause that may impair thyroid regulation. Women with SCH exhibited significantly higher blood pressure and dyslipidemia, confirming the association between mild thyroid dysfunction and cardiovascular risk factors [7]. The elevated total cholesterol and LDL cholesterol levels in the SCH group align with previous studies linking SCH to adverse lipid profiles, potentially increasing the risk of atherosclerosis and coronary artery disease in this vulnerable population. Duration since menopause greater than five years was significantly associated with SCH, suggesting that prolonged estrogen deficiency might contribute to thyroid autoimmunity or functional decline [8]. Although this study did not assess thyroid autoantibodies, this warrants further investigation. The lack of significant differences in clinical symptoms between groups reinforces that SCH often remains asymptomatic, underscoring the importance of routine biochemical screening for early detection [9]. Multivariate analysis confirmed BMI > 27 kg/m^2^ and hypertension as independent predictors of SCH, reflecting the role of metabolic syndrome components in thyroid dysfunction pathogenesis [10]. These findings advocate for integrated management of metabolic health and thyroid function in postmenopausal women [11]. Limitations include the cross-sectional design preventing causal inferences, and absence of thyroid antibody testing which could clarify autoimmune contributions. Future longitudinal studies should explore the progression of SCH and benefits of early intervention in this population. Overall, the study emphasizes the need for thyroid screening and cardiovascular risk assessment in postmenopausal women to optimize health outcomes.

## Conclusion:

Subclinical hypothyroidism is common in postmenopausal women and is significantly associated with higher BMI, hypertension, and longer duration since menopause. It correlates with adverse lipid profiles and increased cardiovascular risk factors, often remaining clinically silent. Routine thyroid function screening in postmenopausal women is essential for early diagnosis and timely management to prevent complications.
